# Platelets in Patients with Premature Coronary Artery Disease Exhibit Upregulation of miRNA340* and miRNA624*

**DOI:** 10.1371/journal.pone.0025946

**Published:** 2011-10-13

**Authors:** Brigitte M. Sondermeijer, Annemieke Bakker, Amalia Halliani, Maurice W. J. de Ronde, Arnoud A. Marquart, Anke J. Tijsen, Ties A. Mulders, Maayke G. M. Kok, Suzanne Battjes, Steffi Maiwald, Suthesh Sivapalaratnam, Mieke D. Trip, Perry D. Moerland, Joost C. M. Meijers, Esther E. Creemers, Sara-Joan Pinto-Sietsma

**Affiliations:** 1 Department of Vascular Medicine, Academic Medical Center, University of Amsterdam, Amsterdam, The Netherlands; 2 Heart Failure Research Center, Academic Medical Center, University of Amsterdam, Amsterdam, The Netherlands; 3 Department of Experimental Vascular Medicine, Academic Medical Center, University of Amsterdam, Amsterdam, The Netherlands; 4 Department of Clinical Epidemiology, Biostatistics and Bioinformatics, Academic Medical Center, University of Amsterdam, Amsterdam, The Netherlands; Leiden University Medical Center, The Netherlands

## Abstract

**Background:**

Coronary artery disease (CAD) is the leading cause of human morbidity and mortality worldwide, underscoring the need to improve diagnostic strategies. Platelets play a major role, not only in the process of acute thrombosis during plaque rupture, but also in the formation of atherosclerosis itself. MicroRNAs are endogenous small non-coding RNAs that control gene expression and are expressed in a tissue and disease-specific manner. Therefore they have been proposed to be useful biomarkers. It remains unknown whether differences in miRNA expression levels in platelets can be found between patients with premature CAD and healthy controls.

**Methodology/Principal Findings:**

In this case-control study we measured relative expression levels of platelet miRNAs using microarrays from 12 patients with premature CAD and 12 age- and sex-matched healthy controls. Six platelet microRNAs were significantly upregulated (miR340*, miR451, miR454*, miR545:9.1. miR615-5p and miR624*) and one miRNA (miR1280) was significantly downregulated in patients with CAD as compared to healthy controls. To validate these results, we measured the expression levels of these candidate miRNAs by qRT-PCR in platelets of individuals from two independent cohorts; validation cohort I consisted of 40 patients with premature CAD and 40 healthy controls and validation cohort II consisted of 27 patients with artery disease and 40 healthy relatives. MiR340* and miR624* were confirmed to be upregulated in patients with CAD as compared to healthy controls in both validation cohorts.

**Conclusion/Significance:**

Two miRNAs in platelets are significantly upregulated in patients with CAD as compared to healthy controls. Whether the two identified miRNAs can be used as biomarkers and whether they are cause or consequence of the disease remains to be elucidated in a larger prospective study.

## Introduction

Coronary artery disease (CAD) is the leading cause of human morbidity and mortality world wide, underscoring the need for innovative diagnostic strategies. Prevention of CAD relies on the accurate identification of individuals at risk of developing CAD. At present CAD risk estimation is based on the assessment of established risk factors using one of the available risk assessment algorithms[1]. However, risk estimates are imprecise in predicting which subjects will develop CAD underscoring the need for innovative diagnostic strategies[2]–[4]. This has prompted the search for novel diagnostics that can improve the identification of individuals at risk of developing CAD. We propose that, in this respect, microRNAs (miRNAs) could be highly useful. MiRNAs are a class of endogenous short (∼22 nucleotides), single-stranded and non-coding RNA molecules, which can affect the expression of many mRNAs. They exert coordinated and potent effects on cell function[5], [6] and are implicated in human diseases[7] including cardiovascular diseases (CVD) [8], [9]. According to current estimates, the human genome is estimated to encode up to 500 miRNAs[10], many of which are expressed in a tissue- and cell-specific manner[11]–[14], making them attractive biomarkers for diagnostic strategies.

Activated platelets play a critical role in the pathophysiology of CAD in the process of acute thrombosis which follows plaque rupture, as well as in chronic plaque formation[15]. This is exemplified by the beneficial effects of anti-platelet therapy in both the acute and chronic phases of the disease. MiRNAs are known to be present in platelets and exert important regulatory functions[16], since platelets also harbour Dicer and Argonaute 2 (Ago2) complexes, which are involved in the processing of miRNA precursors and the control of specific transcripts. Until recently, miRNAs were thought to be present only in tissue and therefore the use of these molecules as biomarkers for CAD would be less practical. It is now clear that miRNAs are also present in the circulation in nucleated blood cells and even in plasma[17], [18] and non-nucleated cells, such as erythrocytes[19] and platelets[16], [20]–[23], making them easily accessible.

Given the importance of platelets in CAD, we hypothesized that platelets from patients manifest different miRNA expression patterns in CAD patients compared to controls. Therefore, we examined the expression levels of platelet miRNAs in patients with CAD and compared them with healthy individuals.

## Methods

### Study populations

#### The array cohort

For the miRNA array study population we selected a cohort of 12 Caucasian male patients with CAD at a young age (premature CAD) and a positive family history of premature cardiovascular disease (CVD), see Definitions S1. These patients are genetically predisposed for CAD leading to a substantial risk for CAD[23]. The patients were selected from the outpatient clinic of the Academic Medical Center (AMC) of Amsterdam, which is specialised in premature CAD. Data collection was done 3.9±2.0 years after the diagnosis of CAD.

The control cohort was composed of 12 healthy Caucasian male volunteers, who were recruited by advertisement and who were matched with the cases for age and smoking habits. Individuals of this control cohort did not have a history of CVD, nor did they have a positive family history of CVD and they were not allowed to use any medication. Patients and controls were excluded when they suffered from diabetes.

### The validation cohorts

To validate the findings of the microarray data, we measured the expression levels of the selected miRNAs in isolated platelets in two independent validation cohorts by qRT-PCR.

#### Validation cohort I

Validation cohort I consisted of 40 premature male CAD subjects and 40 age-matched male controls. Participants were selected in the same way as the population used for the miRNA array analysis, using identical matching inclusion and exclusion criteria. Individuals in the control cohort were again recruited by advertisement. Data collection was done 5.6±3.5 years after the diagnosis of CAD. Inclusion took place from December 2009 to June 2010. Since miRNA expression levels are influenced by medication [24], [25] use we assessed miRNA expression before and after medication use. Therefore, we asked twenty-seven control subjects in this cohort, which are part of a future array study in monocytes, plasma and platelets, to take simvastatin 40 mg once daily for 6 weeks. During the last two weeks acetyl salicylic acid 100 mg once daily was added to the simvastatin. For that matter, they came twice to the clinic.

#### Validation cohort II

Validation cohort II consisted of members of 4 families with high prevalence of premature CAD; 27 atherosclerotic patients and 40 healthy family members. These 4 families were screened at the outpatient clinic. Family members without signs or complaints of CAD underwent a coronary CT-scan to assess subclinical CAD. Cases were defined as having a history of CAD or a coronary calcium score >80^th^ percentile[26]. Controls were defined as having no signs or complaints of CAD and a coronary calcium score <80^th^ percentile.

### Ethics Statement

This study complies with the Declaration of Helsinki. The study protocol was approved by the Medical Ethical Commission of the AMC in Amsterdam and written informed consent was obtained from all subjects.

### Peripheral blood collection and platelet isolation

Non-fasting venous blood samples were drawn without stasis from an antecubital vein, with use of a 19-gauge needle. Blood was collected in 5 trisodium citrate tubes (each 5 ml containing 0.5 ml 0.105 M trisodium citrate, BD Vacutainer). The first sample was discarded. Immediately after blood withdrawal, the samples were centrifuged (180 *g*, 15 min, room temperature, no brake) to obtain platelet-rich plasma (PRP). With a polypropylene pipette, the upper layer of PRP was carefully transferred to a plastic tube to avoid leukocyte contamination. One part of acid–citrate–dextrose (ACD) buffer (0.085 M trisodium citrate, 0.11 M glucose, 0.071 M Citric acid) was added to five parts of PRP and then the PRP was centrifuged (800 *g*, 20 min, room temperature, no brake). The platelet-poor plasma was discarded and the platelet pellet carefully resuspended in Tyrode buffer (136.9 mM NaCl, 2.61 mM KCl, 11.9 mM NaHCO_3_, 5.55 mM Glucose, 2 mM EDTA, pH 6.5). The platelet suspension was centrifuged (800 *g*, 20 min, room temperature, no brake). The supernatant was discarded and the platelet pellet was resuspended in 50 µl sterile phosphate buffered saline (PBS) and stored at −80°C prior to RNA isolation. The isolated platelets were investigated by fluorescence-activated cell sorting (FACS) using monoclonal antibodies against CD45 (BD Biosciences), CD235a (DAKO) and CD61 (BD Biosciences) to identify leukocytes, erythrocytes and platelets. The purity of the isolated platelets was 99.72% by FACS analysis. For information about peripheral blood collection and platelet isolation of the validation cohorts, see Methods S1.

### RNA isolation

Platelet RNA was isolated using the *mir*Vana PARIS kit (Ambion, Inc.), according to the manufacturer's protocol for liquid samples. The protocol was modified such that samples were extracted twice with an equal volume of acid-phenol chloroform and the column was dried for 3 minutes after the last washing step and before elution. Samples were concentrated from 50 µl to 12 µl, of which 5 µl was used for Illumina arrays as described below.

### MiRNA arrays

MiRNA expression profiles were obtained using Illumina Human v2 MicroRNA Beadarrays according to the manufacturer's recommendation (Illumina, Inc., San Diego, CA) at ServiceXS (Leiden, The Netherlands). For detailed information of the MiRNA expression profiling see Methods S1. Raw data were pre-processed, summarized, log-transformed and quantile normalized using the beadarray package (version 1.12.1) in the statistical software package R (version 2.9.0). Differential expression was assessed using a moderated t-test (limma package version 2.18.3). MiRNAs were considered significantly differentially expressed if the P-values, adjusted for multiple testing by using Benjamini and Hochberg's method, were less than 0.05. Differentially expressed miRNAs (adjusted p<0.05) were visualized by hierarchical clustering of the samples (Euclidean distance, complete linkage). All data is MIAME compliant and the raw data has been deposited in the a MIAME compliant database GEO, as detailed on the MGED Society website http://www.mged.org/Workgroups/MIAME/miame.html. For a more detailed description of the microarray analysis, see Methods S1.

### Validating miRNA expression by real-time PCR

The methods for expression analysis by real-time PCR and the miRNA primers sequences are described in the Methods S1.

## Results

### Clinical characteristics

The clinical characteristics of the miRNA array cohort and both the validation cohorts are shown in Table 1. Individuals in *the array cohort* had a mean age of 45.5±6.6 years and did not differ between patients and controls. The average age of onset of CAD in patients was 42±7.6 years. This cohort consisted of 12 controls and 12 selected patients with premature atherosclerosis. Half of the patients presented with a myocardial infarction and half of them with stable angina. The time of data collection was on average 3.9±2.0 years after the diagnosis of CAD.

**Table 1:** Characteristics of the subjects.

	Microarray		Real-Time PCR			
			*Validation cohort I*		*Validation cohort II*	
	Patient n = 12	Control n = 12	Patient n = 40	Control n = 40	Patient n = 27	Control n = 40
Age at data collection. yrs ± SD	45.7±6.5	45.4±7.0	51.4±4.7	51.0±4.6	59.4±9.8	37.7±11.2*
Age at onset of CAD. yrs ± SD	42±7.6	-	44.9±3.8	-	50.5±9.3	-
CAD defined as AMI. n (%)	6 (50)	-	20 (50)	-	8 (30)	-
CAD defined as stable angina. n (%)	6 (50)	-	20 (50)	-	13(48)	-
Time from diagnosis. yrs ± SD	3.9±2.0	-	5.6±3.5	-	8.6±9.9	-
Current Smoking. n (%)	1 (8)	1 (8)	11 (28)	5 (13)	12 (44)	11 (27.5)
Smoking at time of event. yrs ± SD	4 (33)	-	24 (60)	-	18 (66)	-
Packyears. yrs ± SD	16.6±9.7	5.9±3.8	18.7±15.3	5.7±10.2*	24.4±22.3	6.3±9.1*
Hypercholesterolemia. n (%)	2 (17)	-	5 (12.5)	-	4 (14.8)	1 (2.5)
Hypertension. n (%)	2 (17)	-	6 (15)	-	10 (37)	2 (5)*
Acetylsalicylic acid. n (%)	12 (100)	-	34 (85)	-	19 (70)	-
Lipid lowering drugs. n (%)	11 (92)	-	38 (95)	-	19 (70)	2 (5)*
RAAS-inhibitor. n (%)	4 (33)	-	12 (30)	-	12 (44)	2 (5)*
Beta blocker. n (%)	5 (42)	-	26 (65)	-	12 (44)	2 (5)*

Individuals in *validation cohort I* had a mean age of 51.2±4.6 years at data collection and did not differ between patients and controls. Furthermore, the average age of onset of CAD in patients was 44.9±3.8 years. This cohort consisted of 40 selected patients with premature CAD and 40 healthy *age matched* controls. Half of the patients presented with a myocardial infarction and half of them with stable angina. The time of data collection was on average 5 years after the diagnosis of CAD. Smoking was significantly higher among patients as compared to controls.

In *validation cohort II* which was a random sample of families from our outpatient clinic and was the only cohort without a matched control group, individuals had a mean age of 46.6±15.1 years. This cohort was composed of 4 families and consisted of 27 patients and 40 healthy family members. The age of the cases and controls was 59.4±9.8 and 37.7±11.2 respectively. This was significantly different due to the fact that this cohort was composed of subjects from three generations. Furthermore, smoking and a history of hypertension were also significantly different between patients and controls (all; p<0.05). The average age of onset of CAD in patients was 50.5±9.3 years. About 30% of patients presented with a myocardial infarction and 48% had stable angina. The remaining 22% had non-coronary vascular disease. The time of data collection was on average 5 years after the diagnosis of CVD.

### MiRNA profiles of platelets of CAD patients

**Figure 1:**
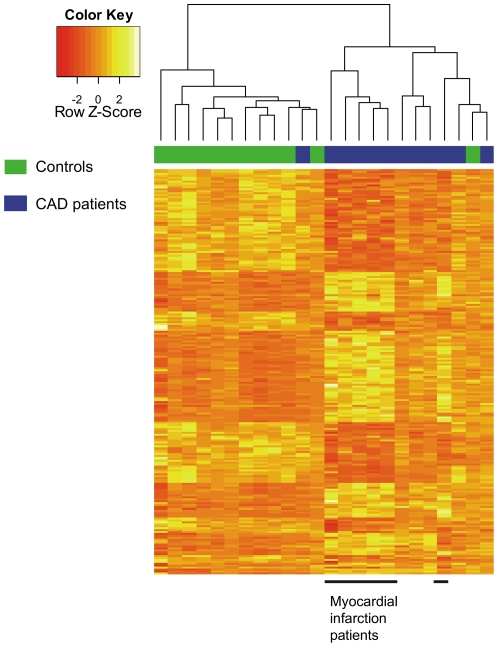
Heatmap. Heatmap of the supervised hierarchical clustering analysis on the expression profiles of the miRNA array analysis of 24 subjects. Subjects are labelled as either controls (green) or cases (blue) at the top of the figure (sample identification on x-axis). Names of the depicted 214 differentially expressed miRNAs (adjusted p<0.05) are listed in a separate table in the same order (see Data S1 online). The key colour bar indicates standardized miRNA expression levels (dark red indicates relatively lower expression; light yellow indicates relatively higher expression).

Each Illumina beadarray contained 1146 human miRNAs as annotated in the Sanger miRBase 12.0 (see Data S1). In total we identified 214 miRNAs, which where differentially expressed in platelets from CAD patients as compared to controls (Data S1). Supervised hierarchical clustering analysis of these 214 miRNAs showed distinct patterns of miRNA expression levels between CAD patients and healthy controls (figure 1). Interestingly, the hierarchical clustering analysis showed differential expression between patients with a myocardial infarction as compared to patients with stable angina. Because of the small number of subjects, this did not reach statistical significance.

**Figure 2:**
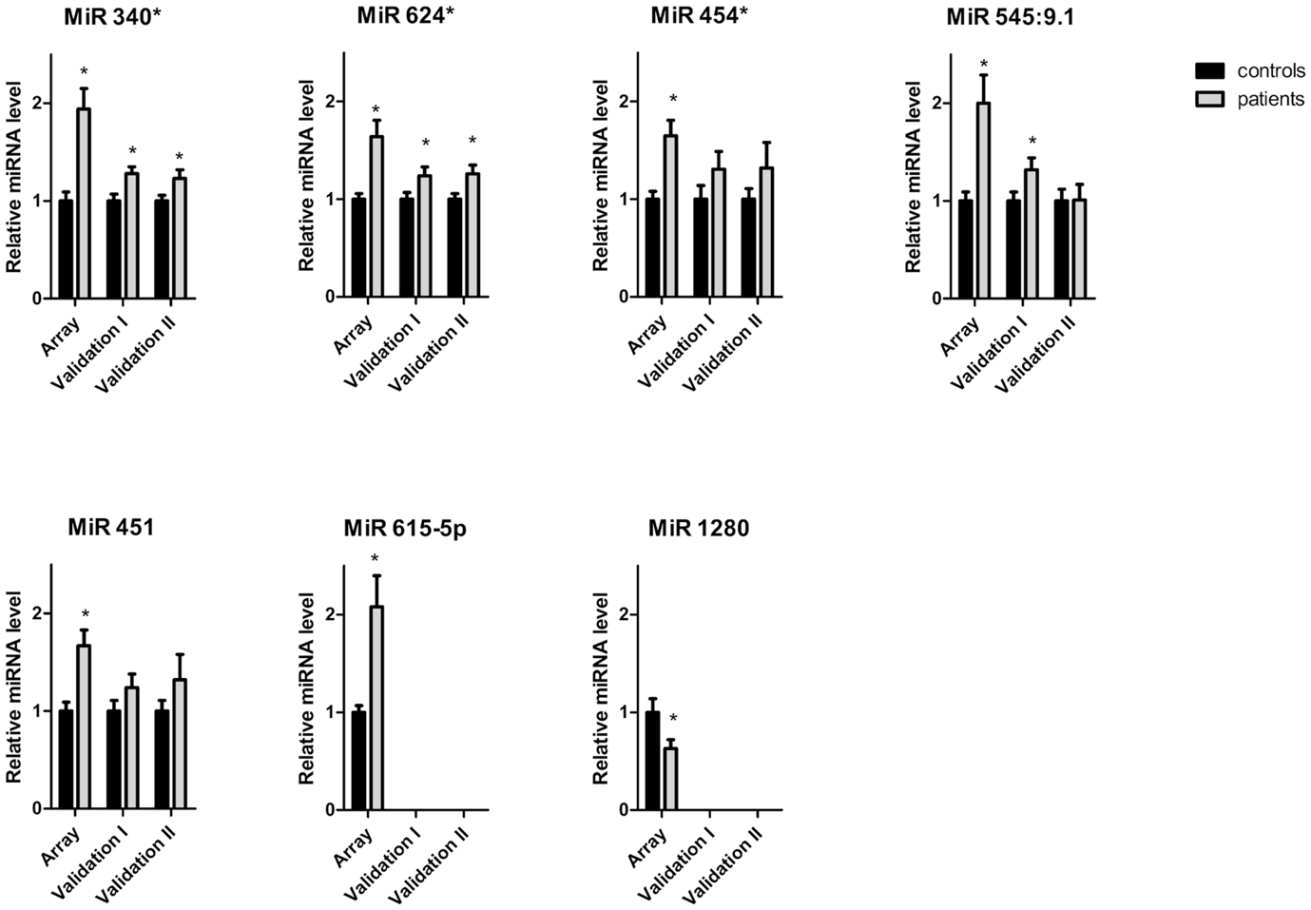
Seven candidate miRNAs expression profiles of platelets from CAD patients and control subjects. Left two bars of each figure represent average miRNA expression of the miRNA array cohort (12 controls and 12 patients). Validation by real-time PCR was performed on two independent cohorts (right four bars). Data are presented as mean±SEM and * indicates p<0.05 compared to healthy controls.

Out of the 214 miRNAs identified in the microarray profiling analysis, 9 miRNAs were at least 1.5-fold upregulated and 4 miRNAs were at least 1.5 fold downregulated in patients as compared to controls. Since the hierarchical clustering data showed 1 patient to cluster within the control group and 1 control subject to cluster within the patient group, we also analysed our data without these 2 subjects, to minimize influences of these 2 misclassified subjects (figure 1, green and blue subject). After all the analysis, six out of 9 miRNAs remained significantly and more than 1.5 fold upregulated (miR340*, miR615-5p, miR545:9.1, miR451, miR454* and miR624*) and 1 out of 5 miRNA remained significantly and more than 1.5 fold downregulated (miR-1280) in both analyses (figure 2).

### Validation of the candidate miRNAs

#### Valdiation cohort I

The 7 differentially expressed miRNAs identified by the microarray analysis were first validated, by real-time PCR, in validation cohort I. Of the 7 miRNAs, 2 miRNAs, miR340* and miR624*, were significantly upregulated in patients as compared to controls (figure 2).

#### Validation cohort II

After the first validation, we validated our results in a second more general cohort of patients of our premature CAD outpatient clinic. Of the 7 miRNAs identified in the array analysis, the same 2 miRNAs; miR340* and miR624*, were significantly upregulated in patients as compared to controls (figure 2).

We encountered technical difficulties in validating 2 of the 7 miRNAs. MiR615-5p and the only downregulated miRNA, miR1280, could not be validated, since the PCR primers for these particular miRNAs did not appear to amplify a specific product.

### Medication use and platelet miRNA expression

To investigate whether medication use might have influenced the miRNA expression levels of our two candidate miRNAs, miR340* and miR624*, we also analysed the expression of these miRNAs. We compared the miRNA expression changes in the platelet miRNA of controls who took medication for 6 weeks compared to the expression levels of controls taking no medication (validation cohort I), as described in the method section; this did not change the results.

### Diagnostic accuracy

When evaluating the combination of 4 candidate miRNAs, miR340* miR624*, miR451 and miR454*, in our largest validation cohort (validation cohort I), that the diagnostic accuracy was AUC = 0.71, 95% CI: 0.59–0.83, p<0.002.

## Discussion

MiRNA array analysis revealed two platelet-derived miRNAs (miR624* and miR340*) to be significantly upregulated in patients with CAD as compared to healthy controls. Besides, these results could be validated in two independent cohorts.

This is the first study that shows an association between platelet miRNAs and patients with CAD. Previous studies in humans have found useful circulating miRNAs as biomarkers for either an acute myocardial infarction[27]–[30] or coronary artery disease[31]–[35]. Concerning myocardial infarction, miR1[27] and miR499[28] were both detected by array analysis of animal cardiac tissue and validated in plasma of patients with an acute myocardial infarction, by qRT-PCR methods. Only recently microarray analysis of miRNAs on peripheral blood or peripheral blood components in relation to cardiovascular disease was performed. The first study by Fichtlscherer et al. [31] investigated plasma miRNA profiles of 8 patients with CAD and 8 healthy controls. They found miR-126, miR-17 and miR-92a (endothelial miRNAs), miR-155 (inflammatory miRNA) and miR-145 (smooth-muscle miRNA) to be downregulated in patients as compared to controls. Furthermore, miR-133a and miR-208a (cardiac-muscle miRNAs) were significantly upregulated. These results could be confirmed in 2 different validation cohorts. The second study investigated whole blood miRNA arrays in relation to an acute myocardial infarction and found miR-1291 and miR-663b to have the highest sensitivity and specificity for the discrimination of cases and controls[29]. In contrast to our study, the above mentioned human studies were performed on plasma or whole blood miRNAs and not on platelet miRNAs [15].

Landry et al.[16] were the first to establish the existence and functionality of miRNA pathway components in platelets of healthy volunteers. Not only did they show that human platelets harbour an abundant and diverse array of miRNAs, further analysis revealed that they also contain the Dicer and Argonaute 2 complexes, which function in the processing of exogenous miRNA precursors and the control of specific reporter transcripts. These findings confirm previous studies reporting the presence of miRNAs in platelet rich plasma, both in healthy donors [22] and patients with polycythemia vera[20]. On the other hand, these observations might have been disturbed by leukocyte contamination. Concerning the leukocyte contamination of our study, we were able to show a platelet purity of 99.72%, therefore contamination seems highly unlikely. When comparing the identified miRNAs of Landry with our list of 30 highest expressed miRNAs of platelets in healthy controls we found that 85% of the miRNAs expressed in their study are in agreement with our study. Moreover, when comparing our 30 highest expressed miRNAs of our platelets in healthy controls with the miRNAs in platelets of healthy subjects found by Hunter et al.[21], who profiled 420 known mature miRNAs by qRT-PCR, we found an overlap of 48%. Considering the technical differences between our study and other studies in profiling methods (Illumina beadchips versus LNA-based microarrays versus qRT-PCR) and RNA isolation methods (*mir*Vana kit versus Trizol), this overlap is rather large, supporting the robustness of our data.

It could be argued that usage of medication in our patient population might have caused the observed differences in miRNA expression levels. We propose that this is not the case, since our validation cohort I was evaluated before and after medication use, which did not influence the results for miR340* and miR624*.

One could speculate which mRNAs are regulated by the two miRNAs identified in this study. We were, however, not able to identify any association between these miRNAs and any disease or tissue involvement.

Regarding the identification of a possible biomarker, the results of our study are limited. The relatively small differences in miRNA expression levels between controls and patients make it unlikely that these miRNAs will serve as independent biomarkers. On the other hand, a combination of low expressing biomarkers might exert good prognostic value. Indeed Meder et al. identified a unique signature of 20 miRNAs that predicted acute myocardial infarction with higher power and accuracy than any single miRNAs [29]. With our analysis of the combination of 4 candidate miRNAs, miR340* miR624*, miR451 and miR454*, we found a diagnostic accuracy of AUC = 0.71, This is in the same range as the diagnostic accuracy of the Framingham risk score (FRS), showing that during a 10-year follow-up the FRS was associated with CVD with an AUC of 0.68 up to 0.77, depending on the investigated population [36].

In conclusion we show that, years after the onset of CAD, different platelet miRNA expression patterns exist between patients and controls. These platelet miRNAs can potentially fine-tune expression of specific gene products that may be involved in governing platelet reactivity. Therefore, a dysfunctional miRNA-based regulatory system could lead to the development of serious platelet-related cardiovascular diseases. Whether the two identified miRNAs can be used as biomarkers and whether they are cause or consequence of the disease remains to be elucidated in a larger prospective study, since the miRNA array profiling took place after the event in all populations.

## Supporting Information

Definitions S1 Definitions with regard to the subjects.
(DOC)

Methods S1 Expanded methods.
(DOC)

Data S1 Names of the depicted 214 differentially expressed miRNAs (adjusted P<0.05). FC: fold change.
(DOC)
