# Supplementary material for: Platelets in Patients with Premature Coronary Artery Disease Exhibit Upregulation of miRNA340* and miRNA624*
Source: PLoS One. 2011 Oct 13;6(10):e25946. doi: 10.1371/journal.pone.0025946 (PMC3192762; doi:10.1371/journal.pone.0025946)
Supplement: Methods S1 — Expanded methods. (DOC) [file pone.0025946.s002.doc]

**Supplementary Methods S1**

**Expanded methods**

*Peripheral blood collection and platelet isolation of validation cohorts.*

Non-fasting venous blood samples were drawn without stasis from an antecubital vein, with use of a 19-gauge needle. Blood was collected in 10 CTAD tubes (each 4.5 ml containing 0.5 ml 0.109 M buffered Citrate, Theophylline, Adenosine and Dipyridamole, BD Vacutainer). Immediately after blood withdrawal, the samples were centrifuged (160*g*, 20 min, room temperature, acceleration 9, brake 1) to obtain platelet-rich plasma (PRP). With a polypropylene pipette, PRP was carefully transferred to a plastic tube leaving at least 1 cm above the interface of the PRP to avoid leukocyte contamination. The number of platelets was determined in a Beckman Coulter counter. To deplete leukocytes, 30 µl Dynabeads CD45 coated beads (4 x 10^8 beads/ml, Invitrogen) were added in phosphate buffered saline (PBS). The PRP samples and the beads were mixed gently on rotary mixer (30 min, room temperature). Mixed PRP and CD45 coated beads were placed on the Magnetic Particle Separator (MPC) for 2 minutes to obtain leukocyte depleted PRP. Leukocyte depleted PRP was transferred to a fresh tube. The number of platelets in leukocyte depleted PRP was determined. The remaining leukocyte depleted PRP was centrifuged 1500g, 20 min, room temperature, acceleration 9, brake 1. The platelet-poor plasma was discarded. The platelet pellet was snap-frozen and stored at -80^o^C before RNA isolation. The number of platelets in the pellet was determined by subtracting platelet count of the platelet-poor plasma from the platelet count of the leukocyte depleted PRP and multiplying it by the original volume of leukocyte depleted PRP. The purity of the isolated platelets in the validation cohorts was determined with Giemsa staining and was >99.60%. Total RNA from platelets was extracted using the *mir*Vana miRNA Isolation kit (Ambion, Inc.) essentially according to the manufacturer’s protocols. The protocol was modified such that the column was air-dried for 3 minutes after the last washing step and before elution.

*MiRNA expression profiling*

A detailed description of the workflow is available at

<http://www.illumina.com/Documents/products/datasheets/datasheet_microrna_expression_profiling.pdf> In short, sample RNA was polyadenylated and then converted to cDNA using a biotinylated oligo-dT primer with a universal PCR sequence at its 5' end, After cDNA synthesis, miRNAs were individually interrogated using specific oligonucleotides. A single miRNA-specific oligo (MSO) is used to assay each miRNA on the panel. All MSOs are hybridized to the sample in parallel, followed by a solid-phase primer extension step. Universal amplification of extended products (universal PCR step) creates fluorescently labeled products identifiable by a unique address sequence. Single-stranded PCR products (750ng) were hybridized to the BeadChip, where the labeled strand binds to the bead on the array containing the complementary address sequence. BeadChips were scanned using the Illumina BeadArray Reader, which measures the fluorescence intensity at each addressed bead location. The Human v2 miRNA panel targets 1146 miRNAs (> 97% coverage of miRBase release 12). Each miRNA is targeted by multiple (~300 on average) identical beads.

*Microarray data analysis*

The raw scan data were read using the beadarray package (version 1.12.1), available through Bioconductor [2]. Illumina’s default pre-processing steps were performed using beadarray. In short, estimated background was subtracted from the foreground for each bead. For replicate beads, outliers greater than 3 median absolute deviations (MADs) from the median were removed and the average signal was calculated for the remaining intensities. Summarized data was log-transformed to ensure that the gene-wise variances are comparable and then quantile normalized [3]. MiRNAs were excluded from the analysis when their log2 expression was less than median expression level on all 24 arrays. Differential expression analysis was performed by fitting a linear model to the 24 arrays and comparing CAD patients and controls using empirical Bayes moderated t-statistics from the Bioconductor software package limma [4]. MiRNAs were considered significantly differentially expressed if the P-values, adjusted for multiple testing by using Benjamini and Hochberg's method, were <0.05. The false discovery rate was thereby controlled to be <5%. Differentially expressed miRNAs (p<0.05) were visualized by hierarchical clustering of the samples (Euclidean distance, complete linkage).

*Expression analysis by quantitative Real-Time PCR.*

To validate results of the microarray analysis, differentially expressed miRNAs between patients and controls were selected for real-time PCR. A fixed volume of 8 μl of the eluate from the RNA isolation was used as input in the reverse transcription reaction. Input RNA was reverse transcribed using the miScript reverse transcription kit (Qiagen) or TaqMan MicroRNA reverse transcription kit (Applied Biosystems). The real-time PCR of miR340*, miR451 and miR624* was performed using High Resolution Melting Master (Roche). The real-time PCR of miR454*, miR545:9.1 and miR615-5p were performed by TaqMan MicroRNA assay (Applied Biosystem) and LightCycler 480 probe master (Roche). Real-time PCRs were performed on a LightCycler480 system II (Roche). MiR340*, miR451 and miR624* were analyzed using LinRegPCR quantitative PCR data analysis software, version 12.3[5]. MiR454* and miR545:9.1 were analysed using LinRegPCR quantitative PCR data analysis software version 12.5. To date, no normalization protocol has been established to normalize and validate the miRNA expression levels. Therefore, after calculation of the N0-values, we normalized our data for both platelet count and a miRNA223 which was similarly expressed throughout all subjects [6]. Results are presented as mean ± SEM using the Statistical Package for the Social Sciences (SPSS) for Windows, version 11.0 (SPSS, Chicago IL). Since the data distribution was not normal, miRNAs were log-transformed. For all of the analyses, a p-value < 0.05 was considered to represent a statistically significant difference.

**References**

1. Hauser ER, Mooser V, Crossman DC, Haines JL, Jones CH et al. Design of the Genetics of Early Onset Cardiovascular Disease (GENECARD) study. American Heart Journal 2003; 145(4):602-613
2. [Gentleman RC](http://www.ncbi.nlm.nih.gov/pubmed?term="Gentleman RC"%5BAuthor%5D&itool=EntrezSystem2.PEntrez.Pubmed.Pubmed_ResultsPanel.Pubmed_RVAbstract), [Carey VJ](http://www.ncbi.nlm.nih.gov/pubmed?term="Carey VJ"%5BAuthor%5D&itool=EntrezSystem2.PEntrez.Pubmed.Pubmed_ResultsPanel.Pubmed_RVAbstract), [Bates DM](http://www.ncbi.nlm.nih.gov/pubmed?term="Bates DM"%5BAuthor%5D&itool=EntrezSystem2.PEntrez.Pubmed.Pubmed_ResultsPanel.Pubmed_RVAbstract), [Bolstad B](http://www.ncbi.nlm.nih.gov/pubmed?term="Bolstad B"%5BAuthor%5D&itool=EntrezSystem2.PEntrez.Pubmed.Pubmed_ResultsPanel.Pubmed_RVAbstract), [Dettling M](http://www.ncbi.nlm.nih.gov/pubmed?term="Dettling M"%5BAuthor%5D&itool=EntrezSystem2.PEntrez.Pubmed.Pubmed_ResultsPanel.Pubmed_RVAbstract) et al. Bioconductor: open software development for computational biology and bioinformatics. [Genome Biol.](javascript:AL_get(this, 'jour', 'Genome Biol.');) 2004;**5**:R80.
3. [Bolstad BM](http://www.ncbi.nlm.nih.gov/pubmed?term="Bolstad BM"%5BAuthor%5D&itool=EntrezSystem2.PEntrez.Pubmed.Pubmed_ResultsPanel.Pubmed_RVAbstract), [Irizarry RA](http://www.ncbi.nlm.nih.gov/pubmed?term="Irizarry RA"%5BAuthor%5D&itool=EntrezSystem2.PEntrez.Pubmed.Pubmed_ResultsPanel.Pubmed_RVAbstract), [Astrand M](http://www.ncbi.nlm.nih.gov/pubmed?term="Astrand M"%5BAuthor%5D&itool=EntrezSystem2.PEntrez.Pubmed.Pubmed_ResultsPanel.Pubmed_RVAbstract), [Speed TP](http://www.ncbi.nlm.nih.gov/pubmed?term="Speed TP"%5BAuthor%5D&itool=EntrezSystem2.PEntrez.Pubmed.Pubmed_ResultsPanel.Pubmed_RVAbstract). A comparison of normalization methods for high density oligonucleotide array data based on variance and bias.[Bioinformatics.](javascript:AL_get(this, 'jour', 'Bioinformatics.');) 2003;**19**:185-93.
4. [Smyth GK](http://www.ncbi.nlm.nih.gov/pubmed?term="Smyth GK"%5BAuthor%5D&itool=EntrezSystem2.PEntrez.Pubmed.Pubmed_ResultsPanel.Pubmed_RVAbstract). Linear models and empirical bayes methods for assessing differential expression in microarray experiments.[Stat Appl Genet Mol Biol.](javascript:AL_get(this, 'jour', 'Stat Appl Genet Mol Biol.');) 2004;**3**:Article3.
5. Ruijter JM, Ramakers C, Hoogaars WM, Karlen Y, Bakker O et al. Amplification efficiency: linking baseline and bias in the analysis of quantitative PCR data. Nucleic Acids Res. 2009;**37**:e45.
6. Merkerova M, Belickova M, Bruchova H. Differential expression of microRNAs in hematopoietic cell lineages. Eur J Haematol 2008; 81(4):304-310.
