# Supplementary material for: Platelets in Patients with Premature Coronary Artery Disease Exhibit Upregulation of miRNA340* and miRNA624*
Source: PLoS One. 2011 Oct 13;6(10):e25946. doi: 10.1371/journal.pone.0025946 (PMC3192762; doi:10.1371/journal.pone.0025946)
Supplement: Data S1 — Names of the depicted 214 differentially expressed miRNAs (adjusted P<0.05). FC: fold change. (DOC) [file pone.0025946.s003.doc]

**Supplementary data S1.**

| Nr | ILMN_Gene | FC | adj.P.Val |
| --- | --- | --- | --- |
| 1 | hsa-miR-154 | 0.63 | 0.0478 |
| 2 | hsa-miR-1280 | 0.63 | 0.0431 |
| 3 | hsa-miR-199a-5p | 0.65 | 0.0394 |
| 4 | hsa-miR-17* | 0.66 | 0.0496 |
| 5 | HS_188 | 0.67 | 0.0429 |
| 6 | hsa-miR-328 | 0.69 | 0.0195 |
| 7 | hsa-miR-376a* | 0.70 | 0.0161 |
| 8 | HS_152 | 0.70 | 0.0216 |
| 9 | hsa-miR-505 | 0.70 | 0.0185 |
| 10 | hsa-miR-376a*:9.1 | 0.72 | 0.0200 |
| 11 | hsa-miR-181a* | 0.73 | 0.0273 |
| 12 | HS_150 | 0.73 | 0.0120 |
| 13 | HS_157 | 0.74 | 0.0063 |
| 14 | hsa-miR-517a | 0.75 | 0.0185 |
| 15 | HS_71.1 | 0.75 | 0.0091 |
| 16 | HS_113 | 0.75 | 0.0352 |
| 17 | hsa-miR-146a* | 0.75 | 0.0161 |
| 18 | hsa-miR-106b | 0.76 | 0.0358 |
| 19 | hsa-miR-886-3p | 0.76 | 0.0031 |
| 20 | hsa-miR-187 | 0.77 | 0.0057 |
| 21 | hsa-miR-1237 | 0.77 | 0.0137 |
| 22 | HS_139 | 0.79 | 0.0167 |
| 23 | HS_19 | 0.79 | 0.0025 |
| 24 | hsa-miR-615-3p | 0.79 | 0.0295 |
| 25 | HS_40 | 0.79 | 0.0105 |
| 26 | hsa-miR-665 | 0.79 | 0.0026 |
| 27 | hsa-miR-1224-3p | 0.80 | 0.0156 |
| 28 | HS_303_b | 0.80 | 0.0125 |
| 29 | HS_194 | 0.81 | 0.0185 |
| 30 | hsa-miR-299-5p | 0.81 | 0.0444 |
| 31 | hsa-miR-371-5p | 0.81 | 0.0087 |
| 32 | hsa-miR-1231 | 0.82 | 0.0026 |
| 33 | HS_275 | 0.82 | 0.0473 |
| 34 | solexa-7534-111 | 0.82 | 0.0111 |
| 35 | hsa-miR-550 | 0.82 | 0.0414 |
| 36 | hsa-miR-551a | 0.82 | 0.0237 |
| 37 | hsa-miR-507 | 0.82 | 0.0057 |
| 38 | hsa-miR-519b-3p | 0.83 | 0.0137 |
| 39 | solexa-3022-299 | 0.83 | 0.0437 |
| 40 | hsa-miR-25* | 0.83 | 0.0137 |
| 41 | hsa-miR-583 | 0.84 | 0.0085 |
| 42 | hsa-miR-548m | 0.84 | 0.0251 |
| 43 | HS_68 | 0.84 | 0.0048 |
| 44 | HS_268 | 0.85 | 0.0057 |
| 45 | hsa-miR-591 | 0.85 | 0.0129 |
| 46 | HS_284.1 | 0.85 | 0.0195 |
| 47 | hsa-miR-513a-5p | 0.86 | 0.0483 |
| 48 | hsa-miR-641 | 0.86 | 0.0060 |
| 49 | hsa-miR-1267 | 0.86 | 0.0185 |
| 50 | hsa-miR-518d-3p | 0.86 | 0.0161 |
| 51 | hsa-miR-218-1* | 0.86 | 0.0127 |
| 52 | hsa-miR-647 | 0.86 | 0.0167 |
| 53 | hsa-miR-525-5p | 0.87 | 0.0031 |
| 54 | hsa-miR-299-3p | 0.87 | 0.0030 |
| 55 | HS_184 | 0.87 | 0.0320 |
| 56 | HS_262.1 | 0.87 | 0.0076 |
| 57 | solexa-9578-86 | 0.87 | 0.0041 |
| 58 | hsa-miR-1243 | 0.87 | 0.0016 |
| 59 | hsa-miR-491-3p | 0.87 | 0.0090 |
| 60 | hsa-miR-138-2* | 0.87 | 0.0160 |
| 61 | hsa-miR-455-3p | 0.87 | 0.0027 |
| 62 | HS_60 | 0.87 | 0.0051 |
| 63 | hsa-miR-1206 | 0.88 | 0.0168 |
| 64 | HS_141 | 0.88 | 0.0380 |
| 65 | HS_177 | 0.88 | 0.0079 |
| 66 | HS_22.1 | 0.88 | 0.0200 |
| 67 | hsa-miR-516a-5p | 0.88 | 0.0471 |
| 68 | hsa-miR-560:9.1 | 0.88 | 0.0362 |
| 69 | hsa-miR-516a-3p.hsa-miR-516b* | 0.89 | 0.0417 |
| 70 | hsa-miR-659 | 0.89 | 0.0031 |
| 71 | hsa-miR-20b* | 0.90 | 0.0118 |
| 72 | solexa-539-2056 | 0.90 | 0.0413 |
| 73 | hsa-miR-661 | 0.90 | 0.0091 |
| 74 | HS_110 | 0.90 | 0.0195 |
| 75 | HS_265.1 | 0.90 | 0.0105 |
| 76 | hsa-miR-744* | 0.90 | 0.0414 |
| 77 | hsa-miR-636 | 0.90 | 0.0498 |
| 78 | hsa-miR-1204 | 0.90 | 0.0119 |
| 79 | hsa-miR-214* | 0.90 | 0.0057 |
| 80 | hsa-miR-614 | 0.90 | 0.0108 |
| 81 | hsa-miR-596 | 0.90 | 0.0105 |
| 82 | hsa-miR-20a | 0.90 | 0.0160 |
| 83 | hsa-miR-367* | 0.91 | 0.0472 |
| 84 | hsa-miR-663 | 0.91 | 0.0394 |
| 85 | hsa-miR-181d | 0.91 | 0.0455 |
| 86 | hsa-miR-943 | 0.91 | 0.0057 |
| 87 | hsa-miR-875-5p | 0.91 | 0.0391 |
| 88 | hsa-miR-646 | 0.91 | 0.0127 |
| 89 | hsa-miR-298 | 0.91 | 0.0185 |
| 90 | hsa-miR-1248 | 0.91 | 0.0217 |
| 91 | hsa-miR-581 | 0.92 | 0.0320 |
| 92 | HS_25 | 0.92 | 0.0200 |
| 93 | hsa-miR-206 | 0.92 | 0.0337 |
| 94 | HS_106 | 0.92 | 0.0113 |
| 95 | hsa-miR-488* | 0.92 | 0.0213 |
| 96 | HS_182.1 | 0.92 | 0.0383 |
| 97 | hsa-miR-548i | 0.92 | 0.0471 |
| 98 | hsa-miR-578 | 0.92 | 0.0185 |
| 99 | hsa-miR-195* | 0.92 | 0.0137 |
| 100 | hsa-miR-106a:9.1 | 0.93 | 0.0248 |
| 101 | hsa-miR-100* | 0.93 | 0.0169 |
| 102 | solexa-15-44487 | 0.93 | 0.0195 |
| 103 | HS_273 | 0.93 | 0.0195 |
| 104 | HS_168 | 0.93 | 0.0442 |
| 105 | HS_23 | 0.93 | 0.0418 |
| 106 | hsa-miR-572 | 0.94 | 0.0206 |
| 107 | hsa-miR-1205 | 0.94 | 0.0325 |
| 108 | hsa-miR-1261 | 0.94 | 0.0325 |
| 109 | HS_79.1 | 0.94 | 0.0435 |
| 110 | HS_89 | 1.06 | 0.0463 |
| 111 | HS_90 | 1.06 | 0.0416 |
| 112 | hsa-miR-934 | 1.06 | 0.0186 |
| 113 | HS_147 | 1.06 | 0.0300 |
| 114 | hsa-miR-1255b | 1.07 | 0.0307 |
| 115 | hsa-miR-548c-3p | 1.07 | 0.0329 |
| 116 | HS_49 | 1.07 | 0.0396 |
| 117 | hsa-miR-1269 | 1.07 | 0.0358 |
| 118 | hsa-miR-1207-3p | 1.08 | 0.0218 |
| 119 | HS_304_b | 1.08 | 0.0090 |
| 120 | HS_267 | 1.09 | 0.0200 |
| 121 | hsa-miR-144 | 1.09 | 0.0166 |
| 122 | HS_14.1 | 1.09 | 0.0237 |
| 123 | hsa-miR-302c | 1.09 | 0.0169 |
| 124 | hsa-miR-1257 | 1.09 | 0.0241 |
| 125 | hsa-miR-634 | 1.09 | 0.0331 |
| 126 | hsa-miR-569 | 1.09 | 0.0444 |
| 127 | HS_9 | 1.09 | 0.0161 |
| 128 | HS_15.1 | 1.09 | 0.0371 |
| 129 | hsa-miR-30b* | 1.10 | 0.0483 |
| 130 | hsa-miR-31* | 1.10 | 0.0186 |
| 131 | hsa-miR-570 | 1.10 | 0.0100 |
| 132 | hsa-miR-346 | 1.10 | 0.0200 |
| 133 | solexa-3464-254 | 1.10 | 0.0394 |
| 134 | hsa-miR-509-5p | 1.10 | 0.0205 |
| 135 | hsa-miR-520b.hsa-miR-520c-3p.hsa-miR-520f | 1.11 | 0.0169 |
| 136 | hsa-miR-155* | 1.11 | 0.0117 |
| 137 | hsa-miR-1200 | 1.12 | 0.0195 |
| 138 | hsa-miR-922 | 1.12 | 0.0325 |
| 139 | hsa-miR-1298 | 1.13 | 0.0137 |
| 140 | hsa-miR-579 | 1.13 | 0.0109 |
| 141 | hsa-miR-541 | 1.13 | 0.0089 |
| 142 | hsa-miR-1258 | 1.13 | 0.0031 |
| 143 | HS_170 | 1.14 | 0.0091 |
| 144 | hsa-miR-648 | 1.14 | 0.0071 |
| 145 | hsa-miR-21 | 1.14 | 0.0130 |
| 146 | hsa-miR-1299 | 1.15 | 0.0279 |
| 147 | HS_95 | 1.15 | 0.0137 |
| 148 | hsa-miR-151-5p | 1.16 | 0.0239 |
| 149 | hsa-miR-147 | 1.16 | 0.0105 |
| 150 | hsa-miR-1247 | 1.17 | 0.0008 |
| 151 | hsa-miR-657 | 1.17 | 0.0234 |
| 152 | hsa-miR-34a* | 1.17 | 0.0064 |
| 153 | hsa-miR-1305 | 1.17 | 0.0315 |
| 154 | HS_99.1 | 1.17 | 0.0057 |
| 155 | hsa-miR-920 | 1.18 | 0.0105 |
| 156 | hsa-miR-325 | 1.18 | 0.0360 |
| 157 | hsa-miR-300 | 1.19 | 0.0017 |
| 158 | hsa-miR-526b | 1.19 | 0.0008 |
| 159 | hsa-miR-563 | 1.19 | 0.0161 |
| 160 | HS_94 | 1.20 | 0.0200 |
| 161 | hsa-miR-455-5p | 1.20 | 0.0017 |
| 162 | hsa-miR-1300 | 1.20 | 0.0034 |
| 163 | hsa-miR-1236 | 1.21 | 0.0012 |
| 164 | hsa-miR-362-3p | 1.21 | 0.0051 |
| 165 | hsa-miR-548g | 1.22 | 0.0011 |
| 166 | hsa-miR-34c-3p | 1.22 | 0.0016 |
| 167 | solexa-7764-108 | 1.22 | 0.0016 |
| 168 | hsa-miR-10a* | 1.22 | 0.0204 |
| 169 | hsa-miR-211 | 1.22 | 0.0113 |
| 170 | hsa-miR-190 | 1.22 | 0.0463 |
| 171 | hsa-miR-1278 | 1.22 | 0.0011 |
| 172 | hsa-miR-429 | 1.23 | 0.0080 |
| 173 | hsa-miR-374a* | 1.23 | 0.0105 |
| 174 | hsa-miR-551b* | 1.23 | 0.0010 |
| 175 | HS_176 | 1.24 | 0.0105 |
| 176 | hsa-miR-504 | 1.24 | 0.0237 |
| 177 | hsa-miR-137 | 1.24 | 0.0233 |
| 178 | hsa-miR-9* | 1.25 | 0.0229 |
| 179 | HS_303_a | 1.25 | 0.0144 |
| 180 | hsa-miR-1283 | 1.27 | 0.0018 |
| 181 | hsa-let-7c* | 1.27 | 0.0125 |
| 182 | hsa-miR-876-5p | 1.27 | 0.0185 |
| 183 | solexa-3277-272 | 1.29 | 0.0103 |
| 184 | hsa-miR-23b | 1.29 | 0.0127 |
| 185 | hsa-miR-374a | 1.29 | 0.0498 |
| 186 | hsa-miR-302c* | 1.29 | 0.0043 |
| 187 | solexa-8926-93 | 1.30 | 0.0142 |
| 188 | HS_86 | 1.30 | 0.0159 |
| 189 | hsa-miR-10b* | 1.32 | 0.0090 |
| 190 | hsa-let-7f-2* | 1.34 | 0.0011 |
| 191 | hsa-miR-557 | 1.35 | 0.0072 |
| 192 | hsa-miR-1245 | 1.36 | 0.0120 |
| 193 | hsa-miR-571 | 1.36 | 0.0169 |
| 194 | hsa-miR-193a-3p | 1.37 | 0.0120 |
| 195 | hsa-miR-182* | 1.38 | 0.0088 |
| 196 | hsa-miR-151-3p | 1.38 | 0.0317 |
| 197 | hsa-miR-651 | 1.38 | 0.0237 |
| 198 | hsa-miR-450b-5p | 1.40 | 0.0251 |
| 199 | HS_122.1 | 1.42 | 0.0099 |
| 200 | hsa-miR-126* | 1.42 | 0.0034 |
| 201 | hsa-miR-10a | 1.42 | 0.0391 |
| 202 | hsa-miR-576-5p | 1.43 | 0.0153 |
| 203 | hsa-miR-132 | 1.43 | 0.0142 |
| 204 | hsa-miR-1289 | 1.46 | 0.0033 |
| 205 | hsa-miR-632 | 1.49 | 0.0024 |
| 206 | HS_65 | 1.54 | 0.0103 |
| 207 | hsa-miR-624* | 1.57 | 0.0137 |
| 208 | hsa-miR-454* | 1.63 | 0.0106 |
| 209 | hsa-miR-451 | 1.63 | 0.0073 |
| 210 | hsa-miR-335* | 1.68 | 0.0397 |
| 211 | hsa-miR-585 | 1.73 | 0.0478 |
| 212 | hsa-miR-545:9.1 | 1.88 | 0.0077 |
| 213 | hsa-miR-615-5p | 1.89 | 0.0071 |
| 214 | hsa-miR-340* | 1.89 | 0.0064 |
